# Exploiting graph kernels for high performance biomedical relation extraction

**DOI:** 10.1186/s13326-017-0168-3

**Published:** 2018-01-30

**Authors:** Nagesh C. Panyam, Karin Verspoor, Trevor Cohn, Kotagiri Ramamohanarao

**Affiliations:** School of Computing and Information Systems, University of Melbourne, Melbourne, Australia

**Keywords:** Relation extraction, Graph kernels, APG kernel, ASM kernel

## Abstract

**Background:**

Relation extraction from biomedical publications is an important task in the area of semantic mining of text. Kernel methods for supervised relation extraction are often preferred over manual feature engineering methods, when classifying highly ordered structures such as trees and graphs obtained from syntactic parsing of a sentence. Tree kernels such as the Subset Tree Kernel and Partial Tree Kernel have been shown to be effective for classifying constituency parse trees and basic dependency parse graphs of a sentence. Graph kernels such as the All Path Graph kernel (APG) and Approximate Subgraph Matching (ASM) kernel have been shown to be suitable for classifying general graphs with cycles, such as the enhanced dependency parse graph of a sentence.

In this work, we present a high performance Chemical-Induced Disease (CID) relation extraction system. We present a comparative study of kernel methods for the CID task and also extend our study to the Protein-Protein Interaction (PPI) extraction task, an important biomedical relation extraction task. We discuss novel modifications to the ASM kernel to boost its performance and a method to apply graph kernels for extracting relations expressed in multiple sentences.

**Results:**

Our system for CID relation extraction attains an F-score of 60%, without using external knowledge sources or task specific heuristic or rules. In comparison, the state of the art Chemical-Disease Relation Extraction system achieves an F-score of 56% using an ensemble of multiple machine learning methods, which is then boosted to 61% with a rule based system employing task specific post processing rules. For the CID task, graph kernels outperform tree kernels substantially, and the best performance is obtained with APG kernel that attains an F-score of 60%, followed by the ASM kernel at 57%. The performance difference between the ASM and APG kernels for CID sentence level relation extraction is not significant. In our evaluation of ASM for the PPI task, ASM performed better than APG kernel for the BioInfer dataset, in the Area Under Curve (AUC) measure (74% vs 69%). However, for all the other PPI datasets, namely AIMed, HPRD50, IEPA and LLL, ASM is substantially outperformed by the APG kernel in F-score and AUC measures.

**Conclusions:**

We demonstrate a high performance Chemical Induced Disease relation extraction, without employing external knowledge sources or task specific heuristics. Our work shows that graph kernels are effective in extracting relations that are expressed in multiple sentences. We also show that the graph kernels, namely the ASM and APG kernels, substantially outperform the tree kernels. Among the graph kernels, we showed the ASM kernel as effective for biomedical relation extraction, with comparable performance to the APG kernel for datasets such as the CID-sentence level relation extraction and BioInfer in PPI. Overall, the APG kernel is shown to be significantly more accurate than the ASM kernel, achieving better performance on most datasets.

## Background

Automated text mining has emerged as an important research topic for effective comprehension of the fast growing body of biomedical publications [1]. Within this topic, relation extraction refers to the goal of automated extraction of relations between well known entities, from unstructured text. Chemical-induced-Disease (CID) relation extraction is motivated by critical applications such as toxicology studies and drug discovery. The importance of CID relations is evident from a recent study of Pubmed search logs [2], that observed that Chemicals, Diseases and their relations are the most popular search topics.

### Relation extraction: sentence vs non-sentence level

A large corpus of annotated Pubmed abstracts for CID relation extraction is now available from BioCreative-V [3] for furthering research and comparison of different methods. This is known as the Chemical-Disease Relations (CDR) corpus. The main objective of the CID relation extraction task defined by BioCreative-V CDR task [3], is to infer Chemical-Disease relations expressed by a Pubmed document (Title and Abstract only). A sample annotated article from this corpus is illustrated in Table 1. More generally, relation extraction from text refers to the task of inferring a relationship between two entities mentioned in the text.

**Table 1:** Illustration of an annotated Pubmed abstract from the CDR corpus

Title	*Propylthiouracil-induced hepatic damage*
Abstract	*Two cases of propylthiouracil-induced liver damage have been observed. The first case is of an acute type of damage, proven by rechallenge; the second presents a clinical and histologic picture resembling chronic active hepatitis, with spontaneous remission.*
Entity	D011441, Chemical, “*Propylthiouracil*”, 0-16
Entity	D011441, Chemical, “*propylthiouracil*”, 54-70
Entity	D056486, Disease, “*hepatic damage*”, 25-39
Entity	D056486, Disease, “*liver damage*”, 79-91
Entity	D006521, Disease, “*chronic active hepatis*”, 246-270
Relation (CID)	D011441 - D006521
Relation (CID)	D011441 - D056486

Within this corpus, many relations may be inferred by analyzing a single sentence that bears the mentions of the relevant entities (Chemical and Disease). We refer to such relations as *sentence level* relations. For example, the relation between “Propylthiouracil” and “hepatic damage” can be inferred by analyzing the single sentence in the title. non-sentence level relations, such as the relation between “propylthiouracil” and “chronic active hepatitis”, are those in which the entity mentions are separated by one or more sentence boundaries. These relations cannot be inferred by analyzing a single sentence. We refer to such relations as the *non-sentence level* relations.

Prior research has shown that relation extraction can be addressed effectively as a supervised classification problem [4], by treating sentences as objects for classification and relation types as classification labels. Classifiers such as Support Vector Machines (SVMs) are typically used for high performance classification by first transforming a sentence into a flat feature vector or directly designing a similarity score (implemented as a kernel function) between two sentences. Kernel methods allow us to directly compute a valid kernel score (a similarity measure) between two complex objects, while implicitly evaluating a high dimensional feature space.

The approach of using a kernel is favored for working with syntactic parses of a sentence which are highly structured objects such as trees or graphs. Tree or graph kernels are known to be efficient in exploring very high dimensional feature spaces via algorithmic techniques. Deep learning [5, 6] based efforts are other alternatives, whose goal is to enable discovery of features (representation learning) with little or no manual intervention. However, we limit our scope in this work, to exploring kernel methods for CID relation extraction. We first illustrate parse structures and then describe the kernels developed for using these parse structures.

### Parse trees vs parse graphs

**Fig. 1:**
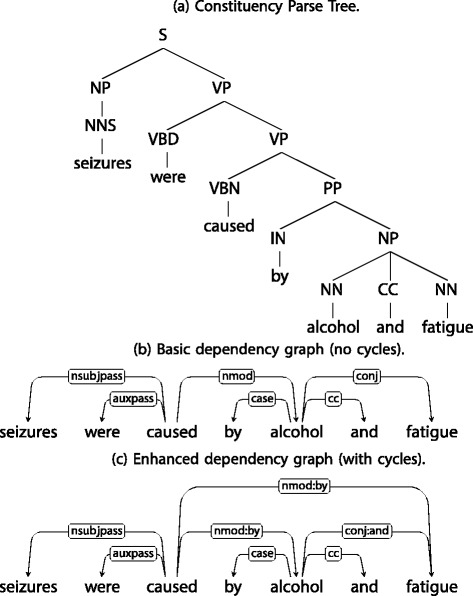
Illustration of different parse structures for the sentence :“Seizures were caused by Alcohol and Fatigue

Simple approaches that use a bag of words model for a sentence, ignore the inherent order within a sentence. However, a sentence can be mapped to an ordered object such as a tree or a graph by using a syntactic parser [7]. We illustrate the syntactic parse structures of a sample sentence in Fig. 1. A constituency parse tree, encodes a sentence as a hierarchical tree, as determined by the constituency grammar. The internal nodes of this tree carry grammatical labels such as “noun phrase (NP)” and “verb phrase (VP)” and the leaf nodes have as labels the words or tokens in the sentence. In contrast, a dependency graph expresses grammatical relationships such as “noun subject (nsubj)” and “verb modifier (vmod)”, as directed and labelled edges between the tokens in the sentence. The nodes of this graph correspond one-to-one with the tokens of the sentence. The undirected version of a dependency graph, obtained by dropping edge directions, may or may not result in a cycle free graph. For example, the basic version of dependency graphs produced by the Stanford Parser [7] is guaranteed to be cycle free, in its undirected form. However, the enhanced dependency parses produced by the Stanford Parser may contain cycles in its undirected form. In the example illustrated in Fig. 1, note the cycle between the nodes “caused” and “fatigue” in the enhanced dependency graph.

### Kernels

In NLP, tree kernels such as the Subset Tree Kernel (SSTK) [8, 9] and Partial Tree Kernel (PTK) [10] have been used effectively for related tasks such as sentence classification [11]. Tree kernels are applied over syntactic parses such as constituency parse or basic dependency parses [12]. These tree kernels cannot handle edge labels directly and therefore transform the original dependency trees to special trees without edge labels, referred to as the Location Centered Tree [13]. A further limitation is that other forms of parses such as enhanced dependency parses which are arbitrary graphs with cycles, cannot be used with tree kernels. This limitation is overcome with graph kernels such as the All Path Graph (APG) [14] kernel that can work with arbitrary graph structures. However, APG kernel is primarily designed to work with edge weighted graphs and requires special transformation of the dependency graphs output by the parser. APG kernel requires the conversion of edge labels into special vertices and it assigns a heuristically determined weight value to the edges. In contrast, the Approximate Subgraph Matching (ASM) kernel is designed to work directly with edge labels in the graph. We present a detailed discussion of the APG and the ASM graph kernels in “APG kernel” and “ASM kernel” sections.

#### Relation to prior work

In this section, we relate and contrast the contributions of this paper with closely related prior work. In our prior work, we proposed a graph kernel based on approximate subgraph matching (ASM) [15]. ASM kernel adopts an approach to graph similarity that is derived from a subgraph isomorphism based event extraction system [16] developed for biomedical relation extraction [17]. In the first step, ASM seeks to match vertices between the two input graphs. Then, the set of all pair shortest paths from the two input graphs are compared, based on the matched vertices. The similarity estimation is based on the counts of edge labels along the shortest path. In our previous work [18], we evaluated the effectiveness of Subtree (STK) and Subset-tree kernels (SSTK) [8, 19] with constituency parse trees for the CID relation extraction task.

In the current work, we introduce a modified form of ASM kernel that incorporates edge weights in the graph. Note that the ASM kernel as presented in prior work [15] considered edge-unweighted graphs only. This ability to incorporate edge weights enables the ASM kernel to positively discriminate between the shortest dependency path between the entities and other paths in the graph, therefore boosting its performance further. For instance, the CID sentence level relation extraction with ASM kernel as reported in [15] is 58%, but improved to 63% in current work. Secondly, we have extended the evaluation for the CID task with other tree kernels namely the Partial Tree Kernel (PTK) [10] and graph kernels ASM and APG [20] with dependency parse graphs.

### Contributions

A summary of the main contributions of this paper are :

- We demonstrate a high performance CID relation extraction system, reaching an F-score of 60.3*%*. This performance is achieved using an effective method for non-sentence relation extraction, by combining multiple sentence level parse structures into larger units, and then applying the kernel methods on the aggregate parse structures. Our system compares favorably with prior art [21], where an ensemble of machine learning methods was used to achieve an F-score of 56% and then boosted to 61.3*%* using task specific post-processing rules. In contrast, our system is a general purpose relation extraction system, that does not employ any task or domain specific rules.
- We present a novel graph kernel, namely the ASM kernel with modifications to incorporate edge weights in the graph. We provide a comparative study of the performance of the ASM kernel with the state of the art tree and graph kernels, over two important biomedical relation extraction tasks, the Chemical-Induced Disease (CID) and the Protein-Protein Interaction (PPI) tasks. We demonstrate that the ASM kernel is effective for biomedical relation extraction, with comparable performance to the state of the art APG kernel on several datasets such as CID-sentence level relations and BioInfer in PPI.
- All software for reproducing the experiments in this paper, including our implementation of the APG and the ASM graph kernels in the Java based Kelp [22] framework, is available in the public domain^1^.

## Methods

In this section, we describe the 3 main kernel methods that are studied in this paper, namely the Tree Kernels [10, 19, 23], the All Path Graph (APG) Kernel and the Approximate Subgraph Matching (ASM) Kernel [15].

### Tree kernels

Tree kernels [8] using constituency parse or dependency parse trees have been widely applied for several relation extraction tasks [13, 18, 24]. They estimate similarity by counting the number of common substructures between two trees. Owing to the recursive nature of trees, the computation of the common subtrees can be efficiently addressed using dynamic programming. Efficient linear time algorithms for computing tree kernels are discussed in [10].

Different variants of tree kernels can be obtained, based on the definition of a tree fragment, namely subtree, subset tree and partial tree. A subtree satisfies the constraint that if a node is included in the subtree, then all its descendents are also included in the subtree. A subset tree only requires, that for each node included in the subset tree, either all of its children are included or none is included in the subtree. A partial tree is the most general tree fragment, which allows for partial expansion of a node, i.e for a given node in the partial tree fragment, any subset of its children nodes may be included in the fragment. Subset trees are most relevant with constituency parse trees, where the inner nodes refer to grammatical production rules. Partial expansion of a grammatical production rule leads to inconsistent grammatical structures. As such, subset trees restrict the expansion of a node to include all of its children or none. For dependency parse trees with no such grammatical constraints, partial trees are more suitable to explore a wider set of possible tree fragments. We experiment with subset tree kernels (SSTK) with constituency parses and partial tree kernels (PTK) with dependency parses and report the results on both. We illustrate the constituency parse tree for a sample sentence in Fig. 1.

Here, we present the formal definition of tree kernels. Let *T*_1_ and *T*_2_ denote two trees and let *F*={*f*_1_,*f*_2_,…} denote the set of all possible tree fragments. Let *I*_*i*_(*n*) be an indicator function that evaluates to 1 when the fragment *f*_*i*_ is rooted at node *n* and 0 otherwise. The unnormalized kernel score is given by:

1 $$ K(T_{1},T_{2}) = \sum_{n_{1} \in N_{T_{1}}} \sum_{n_{2} \in N_{T_{2}}} \Delta (n1,n2)  $$

where $N_{T_{1}}$ and $N_{T_{2}}$ are the sets of nodes of *T*_1_ and *T*_2_ respectively and $\Delta (n1,n2)= \sum _{i=1}^{|F|} I_{i}(n_{1}) I_{i}(n_{2})$.

Efficient algorithms for computing tree kernels in linear time in the average case are presented in [10]. We used the implementation of tree kernels provided in Kelp [22].

### APG kernel

The APG kernel [14] is designed to work with edge weighted graphs. A given dependency graph *G* needs to be first modified, to remove edge labels and introduce edge weights. Let *e*=*l*(*a*,*b*) denote an edge *e* with label *l*, from the vertex *a* to vertex *b*. For every such edge in the original graph, we introduce a new node with label *l* and two unlabeled edges (*a*,*l*) and (*l*,*b*) in the new graph. The APG kernel recommends a edge weight of 0.3 as a default setting for all edges. To accord greater importance to the entities in the graph, the edges along the shortest path between the two entities are given a larger weight of 0.9. This constitutes the subgraph derived from the dependency graph of a sentence. Another subgraph derived from the linear order of the tokens in the sentence is constructed. In this subgraph, *n* vertices are created to represent the *n* tokens in the sentence. The lemma of a token is set as the label of the corresponding node. These vertices are connected by *n*−1 edges, for the *n* tokens from left to right. That is, edges are introduced between token *i* and token *i*+1. These two disconnected subgraphs together form the final edge weighed graph over which the APG kernel operates.

Let *A* denote the adjacency matrix of the combined graph. Let “connectivity” of a path refer to the product of edge weights along the path. Intuitively, longer paths or paths with lesser edge weights, have connectivity closer to 0 and shorter paths or paths with greater edge weights have a connectivity closer to 1. Note that the matrix *A*^*i*^ represents the sum of connectivity of all paths of length *i*, between any two vertices. The matrix *W* is defined as the sum of the powers of *A*, I.e $W=\sum _{i=1}^{\infty }A^{i}$. It is efficiently computed as *W*=(*I*−*A*)^−1^. Therefore, *W* denotes the sum of connectivity over all paths. Any contribution to connectivity from self loops is eliminated by setting *W*=*W*−*I*. Finally, the APG kernel computes the matrix *G*^*m*^=*LWL*^*T*^, where *L* is the label allocation matrix, such that *L*[*i*,*j*]=1 if the label *l*_*i*_ is present in the vertex *v*_*j*_ and 0 otherwise. The resultant matrix *G*^*m*^ represents the sum total of connectivity in the given graph *G* between any two labels. Let $G_{1}^{m}$ and $G_{2}^{m}$ denote the matrices constructed as described above, for the two input graphs *G*_1_ and *G*_2_. The APG kernel score is then defined as :

2 $$ K(G_{1},G_{2}) = \sum_{i=1}^{|L|} \sum_{j=1}^{|L|} G_{1}^{m}\left[l_{i},l_{j}\right] \times G_{2}^{m}\left[l_{i},l_{j}\right]  $$

#### Impact of linear subgraph

We noticed substantially lower performance with the APG kernel when the labels marking the relative position of the tokens with respect to the entities, i.e. labels such as “before”, “middle” and “after” in the linear subgraph are left out. For example, the F-score for AIMed in PPI task drops by 8 points, from 42 to 34%, when these labels are left out. This highlights the importance of the information contained in the linear order of the sentence, in addition to the dependency parse graph.

### ASM kernel

The ASM kernel [15] is based on the principles of graph isomorphism. Given two graphs *G*_1_=(*V*_1_,*E*_1_) and *G*_2_=(*V*_2_,*E*_2_), graph isomorphism seeks a bijective mapping of nodes *M*:*V*_1_⇔*V*_2_ such that, for every edge *e* between two vertices *v*_*i*_,*v*_*j*_∈*G*_1_, there exists an edge between the matched nodes *M*(*v*_*i*_),*M*(*v*_*j*_)∈*G*_2_ and vice versa. The ASM kernel though, seeks an “approximate” measure of graph isomorphism between the two graphs, that is described below. Let *L* be the vocabulary of node labels. In the first step, ASM seeks a bijective mapping *M*_1_:*L*⇔*V*_1_, between the vocabulary and the nodes, such that *M*_1_(*l*_*i*_)=*v*_*j*_,*v*_*j*_∈*V*_1_ when the vertex *v*_*j*_ has the node label *l*_*i*_. To enable this, all nodes in the graph are assumed to have distinct labels. For every missing label *l*_*i*_ in the vocabulary, a special disconnected (dummy) node *v*_*j*_ with the label *l*_*i*_ is introduced. Next, ASM does not seek matching edges between matching node pairs. Instead, it evaluates the similarity of the shortest path between them.

Consider two labels *l*_*i*_,*l*_*j*_. Let *x*,*y* be the vertices in the first graph with these labels respectively. I.e *M*_1_(*l*_*i*_)=*x*,*M*_1_(*l*_*j*_)=*y*and*x*,*y*∈*V*_1_. Let $P_{x,y}^{1}$ be the shortest path between the vertices *x* and *y* in the graph *G*_1_. Similarly, let *x*^′^,*y*^′^ denote the matching vertices in the second graph. I.e *M*_2_(*l*_*i*_)=*x*^′^,*M*_2_(*l*_*j*_)=*y*^′^and*x*^′^,*y*^′^∈*V*_2_. Let $P_{x^{\prime },y^{\prime }}^{2}$ denote the shortest path between the vertices *x*^′^ and *y*^′^ in the graph *G*_2_. The feature map *ϕ* that maps a shortest path *P* into a feature vector is described following the ASM kernel definition below.

The ASM kernel score is computed as:

3 $$ \begin{aligned} K(G_{1},G_{2}) &= \sum_{i=1}^{|L|} \sum_{j=1}^{|L|} \phi\left(P_{x,y}^{1}\right) \cdot \phi\left(P_{x^{\prime},y^{\prime}}^{2}\right) \\ \text{s.t}\ M_{1}(l_{i}) &= x, M_{1}(l_{j}) = y\ \text{and}\ x,y \in V_{1} \\ \text{and}\ M_{2}(l_{i}) &= x^{\prime}, M_{2}(l_{j}) = y^{\prime}\ \text{and}\ x^{\prime},y^{\prime} \in V_{2} \end{aligned}  $$

#### Feature space

The feature space of ASM kernel is revealed by examining the feature map *ϕ* that is evaluated for each shortest path *P*. ASM kernel explores path similarity along 3 aspects, namely structural, directionality and edge labels, as described below. We use the notation *W*_*e*_ to denote the weight of an edge *e*. An indicator function $I_{e}^{l}$ is used to indicate if an an edge *e* has an edge label *l*. Similar to the APG graph, we set the edge weights to 0.9 for edges on the shortest dependency path between two entities and 0.3 for the others.

Structural similarity is estimated by comparing “path lengths”. Note that similar graphs or approximately isomorphic graphs are expected to have similar path lengths for matching shortest paths. Therefore, a single feature $\phi _{\text {distance}}(P) = \prod _{e \in P} W_{e} $, is computed to incorporate structural similarity, where *W*_*e*_ denotes the weight of an edge *e* in the path *P*.

Directional similarity is computed like structural similarity, but unlike structural similarity, edge directions are considered. ASM kernel computes two features, $\phi _{\text {forward edges}} (P) = \prod _{f \in P}W_{f}$ and $\phi _{\text {backward edges}} (P) = \prod _{b \in P}W_{b} $, where *f* and *b* denote a forward facing and backward facing edge respectively, in the path *P*.

Edge directions may themselves be regarded as special edge labels of type “forward” or “backward”. Edge label similarity generalizes the above notion to an arbitrary vocabulary of edge labels *E*. In particular, *E* is the set of dependency types or edge labels generated by the syntactic parser. For each such edge label *l*∈*E*, ASM kernel computes the feature $\phi _{l} (P) = \prod _{e \in P} W_{e}^{I_{e}^{l}} $, where $I_{e}^{l}$ denotes an indicator function that takes a value 1 when the edge *e* has a label *l* and 0 otherwise.

The full feature map *ϕ*(*P*) is the concatenation of the above described features for structural, directionality and edge label similarity. We illustrate this feature map for a sample enhanced dependency graph illustrated in Fig. 1. For the label pair “seizures, fatigue”, the shortest path *P* is through the single intermediate vertex “caused”. For this path, the non-zero features are : *ϕ*(*P*)={*ϕ*_distance_=(0.9)^2^,*ϕ*_forward edge_=0.9,*ϕ*_backward edge_=0.9,*ϕ*_nsubj_=0.9,*ϕ*_nmod:by_=0.9,}.

### Implementation details

We implemented the APG and ASM kernel in the Java based Kelp framework [22]. The Kelp framework provides several tree kernels and an SVM classifier that we used for our experiments. We did not perform tuning for the regularization parameter for SVM, and used the default settings (*C*-Value =1) in Kelp. Dependency parses were generated using Stanford CoreNLP [7] for the CDR dataset. For the Protein-Protein-Interaction task, we used the pre-converted corpora available from [14]. The corpus contains the dependency parse graphs derived from Charniak-Lease Parser, which was used as input for our graph kernels. All software implemented by us for reproducing the experiments in this paper, including the graph kernels APG and ASM implementations are available in a public repository.

## Results

We evaluate the performance of the ASM and APG kernels. We first describe our experimental setup and then discuss the results of our evaluation of the different kernels for relation extraction.

### CID relation extraction

This experiment follows the Chemical-Induced Disease Relation Extraction subtask of [3]. The CDR corpus made available by [3] contains three datasets, namely the training set, development set and the test set. Each dataset contains 500 PubMed documents (title and abstract only) with gold standard entity annotations. More details about this corpus is available at [3]. A sample Pubmed document is illustrated in Table 1.

#### Classifier setup

We build separate relation extraction subsystems for sentence level relations and non-sentence level relations. That is, for any relation (*C*,*D*) in a document (where *C*,*D* denotes a chemical and disease identifier respectively), we search for any single sentence that bears mentions to both the relevant entities *C*,*D*. If such a sentence is found, it is added as an example into sentence level relation extraction subsystem. When no such sentence can be found, such a (*C*,*D*) pair is regarded as a non-sentence relation. For these relations, we retrieve all sentences bearing a mention to either *C* or *D*. All such sentences are paired to form examples for the relation (*C*,*D*). That is, an example for a non-sentence relation (*C*,*D*) is a pair of sentences, one containing the mention of entity *C* and the second containing the mention of entity *D*.

#### Entity focus

Note that a single sentence can carry multiple entity pair mentions, with different relations between then. For example, the sentence “The *hypotensive* effect of *alpha methyldopa* was reversed by *naloxone*”, carries two entity pair mentions, namely “alpha methyldopa, hypotensive” and “naloxone, hypotensive”. The first entity pair is related (alpha methyldopa causes hypotension) whereas the second entity pair is unrelated. Therefore, the above sentence should be suitably processed to extract two different training or testing examples for classification, that serve two different entity-pairs, namely “alpha methyldopa, hypotensive” and “naloxone, hypotensive”. To distinguish between the two cases, we attach special vertices with the labels “Entity1” and “Entity2”, that are connected to the entity-pair in focus, in the given graph.

#### Examples for the classifier

For sentence level relations, we transform each sentence into tree or graph by retrieving its constituency parse tree or dependency parse graph. For non-sentence relation examples, we first retrieve the underlying pair of sentences representing the example and transform each sentence to a tree or graph. The resultant pair of trees or graphs are then connected at the root node, with a special edge labelled “Sentence Boundary”, to result in a single tree or a graph, that can then be input to a tree or graph kernel based classifier. The relations retrieved from the two subsystems for sentence and non-sentence level relations are merged (union) together, to form the final set of retrieved Chemical-Disease relations for the whole PubMed document.

#### Results for the CID task

The CID Relation extraction performance of the different kernels is characterized by measuring the Precision, Recall and F1 measures. These are presented in Table 2 for the CDR dataset. All the measurements listed in Table 2 are based on relation extraction with gold standard entity annotation. Further, we have provided the performance measurements for sentence-level relations only and non-sentence level relations only, which characterizes the performance of our two relation extraction subsystems. The column “All Relations” represents the performance of the final relation extraction system over the full CDR test data, that corresponds to the subtask of BioCreative-V [3].

**Table 2:** Performance measurements for chemical induced disease relation extraction

Method	Sent-Rel. only			Non-Sent-Rel. only			All relations		
	P	R	F	P	R	F	P	R	F
SSTK with CP-Tree	43.1	73.7	54.4	36.9	14.2	20.5	42.5	56.0	48.3
PTK with LCT	42.2	75.3	54.1	30.5	40.1	34.6	39.5	64.8	49.0
APG with Dep. Graphs	*54.7*	80.6	*65.1*	*47.8*	43.8	*45.7*	53.2	*69.7*	60.3
ASM with Dep. Graphs	51.6	*80.8*	63.0	38.8	36.0	37.3	49.0	67.4	56.8
Hybrid (Prior art [21])	-	-	-	-	-	-	*64.9*	49.2	56.0
Hybrid + Rules (Prior art [21])	-	-	-	-	-	-	55.6	68.4	*61.3*

(Key: P,R,F denotes Precision, Recall and F1 score respectively. Sent-Rel. and Non-Sent-Rel. denotes sentence level relations and Non-Sentence level relations respectively. CP-Tree and LCT denote constituency parse tree and location centered tree. Dep. Graph denotes dependency graph. The best performance is highlighted in italicized font)

#### Comparison with prior art

A suitable comparison from prior art is the CID relation extraction system by [21]. Similar to our system, they use gold standard entity annotations and do not employ any external knowledge source or knowledge base. This prior work by [21] consists of a hybrid system or an ensemble of classifiers based on feature-based model, a tree kernel-based model and a neural network model. Their system is designed for sentence level relations only and ignores non-sentence relations. The F-score of this hybrid system is reported to be 56%. To further boost the performance, the authors in [21], propose the use of custom or CID task specific post processing rules, such as associating the Chemical mentioned in the title with the Diseases mentioned in the abstract. These heuristics were found to help boost the performance of their system to 61.3*%*.

In our work, we do not employ any custom heuristics and instead rely on machine learning techniques only. Interestingly, when we removed our subsystem for non-sentence level relation extraction, we observed that our final CID relation extraction performance, drops to 55.7*%* and 54.0*%* respectively, for the APG and ASM kernel based systems. In other words, our final performance of 60.3*%*, is due to the substantial contribution (+5*%* points in F-score), from the non-sentence relation extraction.

To summarize, our main findings from the CID relation extraction task are:

- The APG and ASM graph kernels substantially outperform the tree kernels for relation extraction.
- APG kernel offers the best performance, with an F-score of 65% for sentence level relation extraction, 45% for non-sentence level relation extraction and 60% for the full CID test relations.
- ASM kernel is effective for relation extraction and its performance approaches that of the state of the art APG kernel, with an F-score of 63% for sentence level relation extraction, 37% for non-sentence relations and 57% for the full CID test relations.
- Our system achieves a close to state of the art performance for CID relation extraction (60% vs 61%), without employing heuristics or task specific rules.
- Effective non-sentence level relation extraction provides a substantial boost (+5 points) to the final F-score for our CID relation extraction task.

### Protein-protein interaction extraction

The Protein-Protein Interaction (PPI) extraction task, involves extracting Protein-pairs that interact with each other, from Biomedical Literature. We used the PPI corpora from [25], that consists of 5 datasets, namely AIMed, BioInfer, HPRD50, IEPA and LLL. These are collections of sentences sourced from biomedical publications about protein interactions. The goal of the PPI task is to analyze these sentences, such as “Isolation of human *delta-catenin* and its binding specificity with *presenilin 1*” and extract interacting Protein-pairs such as (*delta-catenin, presenilin 1*). We used the derived version of the PPI corpora [25], that contains sentences together with their Charniak-Lease Parser based tokenization, part of speech tagging and dependency parse in a standardized XML format. The corpus contains the list of protein-pairs in each sentence with a label “True” for interacting pairs and “False” otherwise. We used the dependency parses in the corpus to produce graphs that serve as inputs for our graph kernel with SVM based classification. We experiment with graph kernels, specifically the APG and ASM kernels. From prior work [26], we know that APG kernel substantially outperforms tree kernels for the PPI task. Therefore, our main objective in this experiment is to characterize the performance of the ASM and APG (our implementation) kernels for the PPI task, and contrast these to the state of the art APG kernel based PPI performance.

#### Results for the PPI task

We evaluate our implementation of the APG and ASM kernels in the cross-learning setting, that involves grouping 4 out of the 5 datasets into one training unit and testing on the one remaining dataset. These results are presented in Table 3. We have also listed the state of the art performance measurements for PPI with the APG kernel, as reported in prior art (see Table 3 of [26]).

**Table 3:** Performance measurements for protein-protein interaction extraction

Method	AIMed				BioInfer				HPRD50			
	P	R	F	A	P	R	F	A	P	R	F	A
SOA	*30.5*	77.5	*43.8*	*77.6*	58.1	*29.4*	39.1	69.6	64.2	*76.1*	*69.7*	*84.0*
APG	28.6	*81.6*	42.3	76.8	*68.6*	28.6	*40.4*	69.7	62.3	69.9	65.9	79.7
ASM	26.3	78.0	39.3	72.9	67.2	22.6	33.8	*74.1*	*66.0*	58.3	61.9	76.2
	IEPA				LLL							
	P	R	F	A	P	R	F	A				
SOA.	78.5	*48.1*	*59.6*	*82.4*	*86.4*	*62.2*	*72.3*	*86.4*				
APG	78.2	41.8	54.5	80.2	84.7	57.3	68.3	83.4				
ASM	*82.8*	17.3	28.6	77.7	79.3	28.0	41.4	75.3				

(Key: P,R, F and A denotes Precision, Recall, F score and area under curve respectively. SOA denotes State of the art performance with APG as reported in [26]). The best performance is highlighted in italicized font)

#### Comparison with prior art

The PPI task, is characterized by the measures Precision, Recall and F-score and the AUC or the Area Under the ROC Curve. As indicated in prior art [26], AUC is invariant to the class distribution in the dataset and is therefore regarded as an important measurement to characterize the PPI extraction performance.

To summarize, our findings from the PPI experiment are:

- We expect the AUC measurements for our APG implementation to match that of the APG implementation in prior art (Table 3 of [14]). The AUC measurements are nearly equal for the larger datasets, AIMed and BioInfer, but differ noticeably for the smaller datasets, HPRD50 and LLL. A likely cause for this variation is the differing classifier frameworks (SVMs vs Regularized Least Squares) used in these two experiments.
- Our APG implementation varies substantially with the prior art, in Precision and Recall and moderately in F-score. These measurements are known to be sensitive to parameter setting of the classifier and less dependent on the kernel characteristics itself. However, due to computational costs, we have not performed any parameter tuning in this work.
- ASM kernel outperforms APG for BioInfer (AUC of 74.1 vs 69.6), which is a large dataset. However, APG kernel outperforms ASM by a substantial difference for all the remaining datasets, namely AIMed, HPRD50, IEPA and LLL. We conclude that ASM is outperformed by APG for the full PPI task.

### Statistical significance testing

The CID and PPI relation extraction tasks, considered different measurements, such as F-score and AUC, that are considered relevant for relation extraction task. In terms of classification accuracy, a better comparison of the two kernels can be performed with the McNemar’s test [27]. McNemar’s test estimates the statistical significance for the null hypothesis that the two classifiers are equally accurate. The *P*-values for the null hypothesis, corresponding to different classification tasks, are listed in Table 4. The datasets for which the null hypothesis can be rejected (*P*-value <0.01) are highlighted. This test confirms that the APG and ASM kernels are *significantly* different in classification accuracy, over several large datasets such as AIMed, BioInfer and CID non-sentence relations.

**Table 4:** Statistical significance (McNemar’s) tests for the ASM and APG classifiers, for the null-hypothesis being that the two classifiers are equally accurate and a significance threshold of 0.01

Dataset	Number of examples		Accuracy		*P*-value
	Training	Testing	APG	ASM	
AIMed	11,246	5,834	58.6	53.1	*3.8e-7*
BioInfer	7,414	9,666	77.8	76.8	*0.0011*
HPRD50	16,647	433	70.9	68.1	0.999
IEPA	16,263	817	73.6	66.5	*3.9e-6*
LLL	16,750	330	75.4	65.1	*2.2e-6*
CID: Sentence level relations.	9,913	5,099	72.2	71.2	0.0969
CID: Non Sentence level relations	21,656	11,562	84.9	84.1	*0.0002*

*P*-values less than the threshold are shown in italicized font

## Discussion

In this section, we present a detailed comparison of the two graph kernels, namely ASM and APG kernel. We focus our study on the graph kernels only as we saw above for CID relation extraction, that they substantially outperform the tree kernels. Interestingly, both kernels follow the approach of comparing node pairs between two graphs to estimate graph similarity. However, the key difference between the two kernels is their treatment of edge labels in the graph. In APG, edge labels in the graph are transformed into intervening nodes. Therefore, the node label vocabulary in APG is a heterogeneous set which is the union of the vocabulary of word lemmas, *V* in the corpus and the vocabulary of edge labels *D* defined by the dependency parser [7]. That is, the set of node labels considered by APG kernels is *L*=*V*∪*D*. The features explored by the APG kernel can be indexed by pairs of node labels, of the form (*V*∪*D*)×(*V*∪*D*).

In ASM kernel, edge labels (dependency types *D*) and node labels (word lemmas *V*) are treated separately. ASM associates a node label pair with a rich feature vector, where each feature is a function of the edge labels along the shortest path between the nodes. Therefore, its feature space can be indexed by triplets of the form (*V*×*V*×*D*). This is an important difference from the APG kernel, which associates a single scalar (graph connectivity) value with a node label pair. The higher feature space dimensionality for the ASM kernel is a likely cause for its lower performance than the APG kernel. The other main difference between the two kernels is that the APG kernel considers all possible paths between a pair of nodes, whereas ASM kernel considers only the shortest path. This is another likely factor, that is disadvantageous to ASM kernel in comparison to APG kernel.

### Error analysis

We manually examined a few error samples to identify the likely causes of errors by APG and ASM kernel in CID and PPI relation extraction tasks. We noticed that an important characteristic of the CDR dataset, which is the presence of many entity pairs in a single sentence, to be a likely cause for the high false positive rate. Consider the example: “Susceptibility to *seizures* produced by *pilocarpine* in rats after microinjection of *isoniazid* or *gamma-vinyl-GABA* into the substantia nigra.” Here, *pilocarpine* and *seizures* are positively related, which is correctly recognized by our classifiers. However, our classifiers also associate the disease *seizures* with the chemicals *isoniazid* and *gamma-vinyl-GABA*. The graph examples corresponding to different entity pairs arising out of the above sentence, share many common subgraph and are likely to be close enough in the feature space of the classifiers. We hypothesize that a sentence simplification step, that trims the sentences into shorter phrases specific to entity-pairs, or a specific treatment of coordination structure in the sentences [28], is likely to reduce the error rates.

Another source of errors is in preprocessing. Consider the following sentence from the PPI corpora: “We took advantage of previously collected data during a randomized double-blind, placebo-controlled clinical trial to conduct a secondary analysis of the *RBP/TTR* ratio and its relationship to infection and VA status.”. In cases like these, the tokenization offered as part of the PPI corpora recognizes the string “RBP/TTR” as a single token. This error in preprocessing causes the corresponding dependency graph to have a single node with the label “RBP/TTR”, instead of two different nodes, corresponding to the proteins “RBP” and “TTR”. Improving preprocessing accuracy is likely to improve the relation extraction performance for PPI.

### Future work

#### Enriching edge labels

The main strength of ASM kernel is that it handles edge labels distinctly from node labels in the graph. This strength can be exploited by designing informative features for edges or paths, that are representative of the corresponding sub-phrase in the sentence, for example, phrase level measurements of sentiment polarity, “negation” and “hedging” [29].

#### Custom edge similarity

ASM computes the similarity of shortest paths, based on their edge label composition. As the dependency edge labels have well defined semantics, designing custom similarity measures between these edge labels is likely to improve performance. These edge labels are grouped in a well defined hierarchical fashion, which the similarity function can exploit. For example, the edge labels “vmod” (verb modifier) and “advmod” (adverbial modifier) are more closely related to each other than to the edge label “nsubj” (nominal subject).

#### Semantic matching

ASM relies on comparing shortest paths between two input graphs, whose start and end nodes have identical labels. Currently, node labels are set to be word lemmas instead of tokens, to improve generalization and address minor variations such as “cured” and “curing”. In future, we aim to explore setting node labels to word classes that group words with similar meanings together. For example, node labels may be set to cluster ids, post word clustering. Semantic matching of lemmas using distributional similarity [30], may allow matching different lemmas with similar meanings (For example, lemmas such as “cure” and “improve”). Similar approaches to tree kernels [31] has been shown to improve performance.

## Conclusion

We demonstrated a method for extracting relations that are expressed in multiple sentences, to achieve a high performance Chemical-Induced Disease relation extraction, without using external knowledge sources or task specific heuristics. We studied the performance of state of the art tree kernels and graph kernels for two important biomedical relation extraction tasks, namely the Chemical-Induced Disease (CID) relation extraction and Protein-Protein-Interaction (PPI) task. We showed that the Approximate Subgraph Matching (ASM) kernel is effective and comparable to the state of the art All Path Graph (APG) kernel, for CID sentence level relation extraction and PPI extraction from BioInfer dataset. The difference in performance between the two kernels is not significant for CID sentence level relation extraction. However, for the full CID relation extraction and most other datasets in PPI, ASM is substantially outperformed by the APG kernel.

## Abbreviations

ASM Kernel: Approximate subgraph matching kernel
APG Kernel: All path graph kernel
CID: Chemical-Induced-Disease
PTK: Partial tree kernel
SSTK: Subset tree kernel
SVM: Support vector machine

## Footnotes

1
https://bitbucket.org/readbiomed/asm-kernel
